# NANOG confers resistance to complement-dependent cytotoxicity in immune-edited tumor cells through up-regulating CD59

**DOI:** 10.1038/s41598-022-12692-6

**Published:** 2022-05-23

**Authors:** Sung Wook Son, Eunho Cho, Hanbyoul Cho, Seon Rang Woo, Hyo-Jung Lee, Se Jin Oh, Suyeon Kim, Jae-Hoon Kim, Eun Joo Chung, Joon-Yong Chung, Min Gyu Kim, Kwon-Ho Song, Tae Woo Kim

**Affiliations:** 1 Department of Cell Biology, Daegu Catholic University School of Medicine, Daegu, 42472 South Korea; 2 Department of Biochemistry and Molecular Biology, Korea University College of Medicine, Seoul, 02841 South Korea; 3 Department of Biomedical Science, Korea University College of Medicine, Seoul, 02841 South Korea; 4 BK21 Graduate Program, Department of Biomedical Science, Korea University College of Medicine, Seoul, 02841 South Korea; 5 Department of Obstetrics and Gynecology, Gangnam Severance Hospital, Yonsei University College of Medicine, Seoul, 06273 South Korea; 6 Radiation Oncology Branch, Center for Cancer Research, National Cancer Institute, National Institutes of Health, Bethesda, MD 20892 USA; 7 Molecular Imaging Branch, Center for Cancer Research, National Cancer Institute, National Institutes of Health, Bethesda, MD 20892 USA; 8 School of Medicine, The Catholic University of Korea, Seoul, South Korea; 9 NEX-I Inc., Seoul, South Korea

**Keywords:** Cancer, Immunology, Molecular biology

## Abstract

Cancer immunoediting drives the adaptation of tumor cells to host immune surveillance. Previously, we have demonstrated that immunoediting driven by cytotoxic T lymphocytes (CTLs) enriches NANOG^+^ tumor cells with immune-refractory properties. Here, we found that CTL-mediated immune pressure triggered cross-resistance of tumor cells to the complement system, a part of the innate immune system. In this process, NANOG upregulated the membrane-bound complement regulatory protein (mCRP) CD59 through promoter occupancy, thereby contributing to the resistance of tumor cells against complement-dependent cytotoxicity (CDC). Notably, targeting of NANOG sensitized the immune-refractory tumor cells to trastuzumab-mediated CDC. Collectively, our results revealed a possible mechanism through which selection imposed by T-cell based immunotherapy triggered complement-resistant phenotypes in the tumor microenvironment (TME), by establishing a firm molecular link between NANOG and CD59 in immune-edited tumor cells. We believe these results hold important implications for the clinical application of CDC-mediated therapeutic antibody.

## Introduction

In the tumor microenvironment (TME), immunological components, including immune cells, adaptive immune cells, and extracellular immune factors, have been shown to be strongly related to tumor development and recurrence^1^. There is increasing evidence to suggest that cancer immunoediting drives the adaptation of tumor cells to host immune surveillance, thereby contributing to the generation of cancer cells with better survival advantages^2^. Several studies have revealed that immune selection imposed by host immune surveillance is also closely linked to the emergence of tumor cells that are refractory to multiple clinical interventions, including chemotherapy, radiotherapy, as well as immunotherapy^3–5^. Therefore, it is necessary to improve our understanding of a complex interplay between cancer cells and immunological components during cancer immunoediting in order to develop successful anti-cancer therapies.

The complement pathway is the first line of defense in the human immune system, and it acts as a key system for immune surveillance^6^. Normal cells are protected from inappropriate complement attack by membrane-bound complement regulatory proteins (mCRPs), including CD46, CD55, and CD59, which prevent complement activation or block the formation of the terminal cytolytic membrane attack complex (MAC)^7^. These mCRPs have been reported to be up-regulated in multiple cancer cell lines and cancer patients, in order to evade immune surveillance and complement-dependent cytotoxicity (CDC)^8^. As complement and antibodies are interdependently correlated with the activation of immune response, inhibited complement activity is not only confined to complement itself, but it also affects the subsequent action of anti-tumoral therapeutic antibodies^9^. Indeed, accumulating evidence indicates that mCRPs also confer tumor cell resistance to antibody-based cancer therapy, such as rituximab and cetuximab induced complement activation^10,11^. Although hyper-expression of mCRPs on the surface of tumor cells is closely linked to cell protection from complement attack during immune surveillance as well as antibody-based cancer therapy, molecular mechanism for the regulation of mCRPs in the course of cancer immunoediting remains unclear.

In an effort to elucidate the molecular mechanisms underlying cancer immunoediting, we have previously found that cytotoxic T lymphocyte (CTL)-mediated immune selection drives the evolution of tumor cells toward an immune-resistant and stem-like phenotype^12,13^. Interestingly, the immune-edited tumor cells were refractory to apoptotic death by multiple therapeutics, including cisplatin, γ-radiation, as well as cognate CTLs, whereas the parental cells remained sensitive to them^3,4,14,15^. In this respect, we have previously demonstrated that NANOG is a key transcriptional factor (TF) driving multi-modal resistance and stem-like phenotype of the immune-refractory tumor^13,16^. However, the functional association between NANOG and complement resistance in CTL-mediated immune editing remains largely unknown.

In this study, we demonstrated a crucial role of NANOG at the crossroads between CTL-mediated immune editing and complement resistance by identifying CD59 as a novel NANOG transcriptional target. Therefore, we have provided proof of the principle that NANOG inhibition is an effective strategy to control human cancer, particularly in the context of antibody-based therapy.

## Results

### CTL-mediated immune selection confers resistance to CDC via up-regulating mCRPs

**Figure 1:**
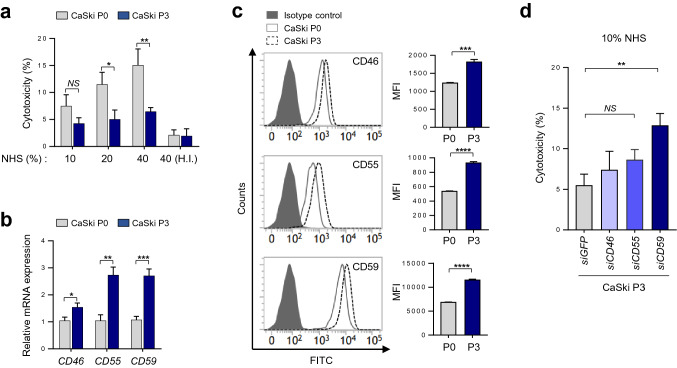
CTL-mediated immune selection confers resistance to CDC via up-regulating mCRPs. (**a**) Complement-dependent cytotoxicity (CDC) of CaSki P0 or P3 cells was measured by the Lactate dehydrogenase (LDH) release assay after incubation with the indicated concentrations of normal human serum (NHS). The heat-inactivated (H.I.) NHS served as negative control. (**b**) The mRNA expression of mCRPs, such as CD46, CD55, and CD59, in CaSki P0 or P3 cells was analyzed by real-time quantitative RT-PCR. (**c**) Protein levels of mCRPs were determined by staining with CD46, CD55, or CD59, followed by flow cytometry analysis. Data are presented as the mean fluorescence intensity (MFI). (**d**) CaSki P3 cells were transfected with siRNAs targeting GFP, CD46, CD55, or CD59. CDC of the transfected cells was measured by the LDH release assay. All experiments were performed in triplicate, and error bars represent standard deviations from the mean. Differences in cytotoxicity or expression level were statistically tested using two-way ANOVA (**a**,**b**), the Student’s t-test (**c**) or one-way ANOVA (**d**): *p < 0.05; **p < 0.01, ***p < 0.001, ****p < 0.0001, *NS*, not significant.

Previously, we established an immune-edited tumor cell line, CaSki P3 (termed P3), generated from its CTL-susceptible parental cell line, CaSki P0 (termed P0), through three rounds of in vitro selection by cognate CTLs^17^. To investigate whether immune pressure imposed by CTLs contributes to CDC resistance of tumor cells, we compared the susceptibility of CaSki cells, before (P0) or after (P3) CTL selection, to normal human serum (NHS)-mediated CDC. We found that CTL-mediated immune selection drove tumor cells to become refractory to NHS, indicating cross-resistance of the CTL-resistant CaSki P3 cells to CDC (Fig. 1a). Increased expression levels of mCRPs were reported to affect the CDC-refractory property of tumor cells^18^. In this respect, we observed increased expression levels of mCRPs, such as CD46, CD55, and CD59, in P3 tumor cells over the course of CTL-mediated immune editing (Fig. 1b,c). To directly link these mCRPs to the CDC-refractory phenotypes of CaSki P3 cells, we silenced the CD46, CD55, CD59 in CaSki P3 cells using siRNA, and found that each siRNA targeting indicated genes exhibited similar knockdown efficiencies on expression of target proteins, such as CD46, CD55, or CD59 (supplementary Fig. S1). Interestingly, CaSki-P3 cells transfected with siRNAs targeting CD59 were more susceptible to NHS-mediated CDC compared with siGFP-transfected P3 cells, whereas CaSki-P3 cells transfected with siCD46 and CD55 did not significantly alter the susceptibility to NHS-mediated CDC (Fig. 1d). The data indicate a crucial role of CD59 in the CDC-refractory property. Taken together, our data indicate that mCRPs, such as CD46, CD55, or CD59, are increased during CTL-mediated immunoediting, and they, especially CD59, contribute to CDC resistance of immune-edited tumor cells.

### NANOG promotes CDC resistance of tumor cells during CTL-mediated immune selection

**Figure 2:**
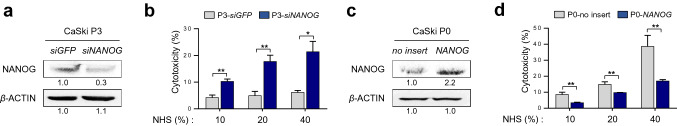
NANOG contributes to CDC resistance of tumor cells. (**a**,**b**) CaSki-P3 cells were transfected with siRNAs targeting GFP or NANOG. (**c**,**d**) CaSki P0 cells were stably transfected with an empty vector (no insert) or NANOG. (**a**,**c**) The level of NANOG protein was analyzed by Western blot. *β*-ACTIN was included as an internal loading control. Numbers below blot images indicate the quantitative value, as measured by fold change. Original blots were presented in Supplementary Fig. S7 and Fig. S8 (**b**,**d**) CDC was measured by LDH release assay after incubation with the indicated concentrations of NHS. All experiments were performed in triplicate, and error bars represent standard deviations from the mean. Differences in cytotoxicity level were statistically tested using the Student’s t-test (**a**,**c**), or two-way ANOVA (**b**,**d**): *p < 0.05; **p < 0.01.

Previously, we demonstrated that NANOG is a key TF driving multi-modal resistance in immune-edited P3 tumor cells^4,14^. As NANOG was upregulated upon CTL-mediated immune selection^14^, we questioned whether NANOG is required for cross-resistance to CDC in P3 cells. Interestingly, silencing of NANOG in P3 cells led to a significant increase in the sensitivity of P3 cells to CDC (Fig. 2a,b), suggesting a crucial role of NANOG in the CDC-refractory property of P3 cells. We then asked whether NANOG expression could induce the CDC-refractory phenotype in P0 cells. Notably, delivery of NANOG to CaSki P0 cells reduced the sensitivity to NHS-mediated CDC (Fig. 2c,d). Based on these data, we conclude that NANOG is a critical mediator that could promote the CDC-refractory property in tumor cells encountering CTL-mediated immune selection.

### NANOG directly regulates CD59 through promoter occupancy

**Figure 3:**
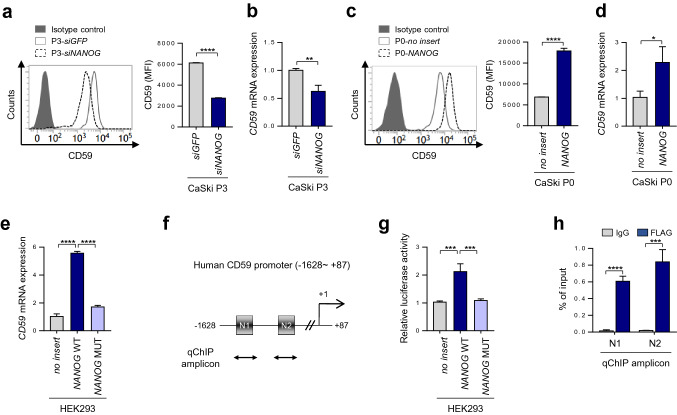
NANOG directly regulates CD59 expression through promoter occupancy. (**a**,**b**) CaSki-P3 cells were transfected with siRNAs targeting GFP or NANOG. (**c**,**d**) CaSki P0 cells were stably transfected with an empty vector (no insert) or NANOG. (**a**,**c**) Protein levels of CD59 were determined by flow cytometry analysis. Data are presented as the mean fluorescence intensity (MFI). (**b**,**d**) The mRNA expression of CD59 was analyzed by real-time quantitative RT-PCR. (**e**) HEK293 cells were transfected with an empty vector (no insert), NANOG wild-type (WT), or NANOG mutant (MUT). The mRNA expression of CD59 in the transfected cells was analyzed by real-time quantitative RT-PCR. (**f**) Diagram of the CD59 promoter region (-1628 to + 87) that contains two NANOG binding elements, indicated by N1 and N2, respectively. Arrows indicate qChIP amplicon corresponding to N1 and N2. (**g**) Luciferase assay in HEK293 cells transfected with the pGL3-CD59 plasmid, together no insert, NANOG WT or NANOG MUT plasmids. A vector expressing β-galactosidase was co-transfected to ensure the transfection efficiency and normalize the luciferase activity values. For analysis of the promoter activity, luciferase activity was normalized to control cells, transfected with no insert plasmid. (**h**) The cross-linked chromatin from HEK 293 cells transfected with FLAG-NANOG was immunoprecipitated with mouse IgG or anti-FLAG antibodies. Relative enrichment of FLAG-NANOG on the CD59 promoter region was assessed by qChIP-PCR analysis with primers that amplify the genomic region indicated by N1 and N2, respectively. The value of ChIP data represents relative ratio to the input. All experiments were performed in triplicate, and error bars represent standard deviations from the mean. Differences in experimental values were statistically tested using the Student’s t-test (**a**–**d**)**,** two-way ANOVA (**h**) or one-way ANOVA (**e**,**g**): *p < 0.05; **p < 0.01, ***p < 0.001, ****p < 0.0001.

Among the mCRPs upregulated upon CTL-mediated immune selection, CD59 had dominant effects on the CDC-resistant phenotype (Fig. 1d); hence, we attempted to elucidate the underlying mechanism responsible for CD59 upregulation in CTL-resistant tumor cells. In this respect, the present study showed that CD59 was upregulated at the transcriptional level during CTL-mediated immunoediting (Fig. 1b). Since NANOG is a key factor driving the CDC-refractory phenotype of P3 cells (Fig. 2), we reasoned that transcriptional activation of the CD59 gene might be dependent on NANOG expression. When we silenced NANOG in P3 cells, the level of surface CD59 was significantly decreased, which was accompanied by decreased mRNA expression of the CD59 (Fig. 3a,b). Conversely, when NANOG was overexpressed in P0 cells, levels of both CD59 protein and mRNA were increased (Fig. 3c,d). These data indicate that NANOG facilitates CD59 transcription during CTL-mediated immune editing.

We next investigated whether CD59 expression is directly regulated by transcriptional function of NANOG. To address this, a mutant of NANOG (NANOG E264G, E268G, E272A, NANOG MUT), which has weak transcriptional activity^13^, and wild type NANOG (NANOG WT) were transfected into HEK293 cells. Consistent with the result in CaSki P0 cells (Fig. 3d), NANOG WT profoundly increased the expression level of CD59 mRNA (Fig. 3e). However, NANOG MUT had no significant impact on CD59 expression (Fig. 3e), indicating that NANOG-mediated CD59 regulation is dependent on the transcriptional activity of NANOG. We further identified the CD59 promoter region containing two NANOG-binding elements (N1 and N2), suggesting that NANOG might be a direct transcriptional activator of CD59 (Fig. 3f). Notably, the promoter activities of CD59 were increased upon NANOG WT but not upon NANOG MUT (Fig. 3g). Furthermore, quantitative ChIP (qChIP) assays showed that NANOG directly bound to its binding elements on the CD59 promoter region (Fig. 3h). Taken together, these results demonstrate that NANOG directly regulates CD59 transcription by binding to the promoter region of the CD59 gene.

### The NANOG-CD59 axis is conserved across various human cancer types

**Figure 4:**
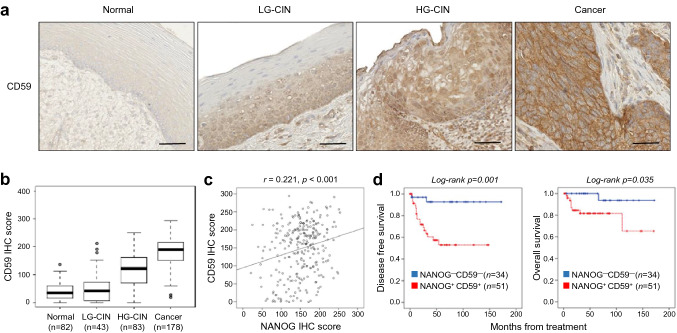
NANOG and CD59 expression in human cervical neoplasia specimens. (**a**) Representative image of immunohistochemical staining of CD59 in the cervical tissue from normal, low grade CIN (LG-CIN), high grade CIN (HG-CIN), and cervical carcinoma (cancer) patients. Scale bar shown is 100 µm. (**b**) Box plot depiction of immunohistochemical staining data shows that the CD59 level was increased during tumor progression. Differences in protein expression were statistically tested using the Kruskal–Wallis test. (**c**) NANOG staining score (IHC score) was compared with CD59 IHC score using a simple scatter plot. NANOG was positively correlated with CD59 (Spearman’s *rho* = 0.221, *p* < 0.001). (**d**) Patient with the combined NANOG^+^/CD59^+^ level showed a significantly worse disease-free survival and overall survival than patients with the NANOG^-^/CD59^-^ level (log-rank test, *p* = 0.001 and *p* = 0.035, respectively). The cut-off value of NANOG and CD59 were 160 and 178, respectively.

We have previously reported that high level of NANOG was correlated with tumor progression and poor outcome of patients with cervical cancer^13,19^. To determine the clinical relevance of the NANOG-CD59 axis in human cancer, we evaluated the CD59 level by immunohistochemistry in cervical tissue specimens from patients with cervical intraepithelial neoplasia (CIN) (Supplementary Table S1) and further analyzed their relationship with the previously reported NANOG^19^. We found that the levels of CD59 were increased as the tumor progressed from normalcy to cancer (Fig. 4a,b). Consistent with our in vitro experimental results, the level of CD59 showed a positive correlation with the level of NANOG (*Spearman’s rho* = 0.221, *p* < 0.001) (Fig. 4c). We next examined the relationship of each protein level with patient survival outcomes. Kaplan–Meier plots demonstrated that patients with a high level of CD59 (CD59^+^) showed shorter disease-free survival than patients with a low level of CD59 (CD59^-^) (Supplementary Fig. S2). Notably, patients with a combined NANOG^+^/CD59^+^ level displayed a significantly worse disease-free survival and overall survival than patients with NANOG^-^/CD59^-^ (Fig. 4d). Previously, we defined a NANOG signature to acquire a more reliable readout indicating NANOG expression in tumor cells^4^. Comparative transcriptome analysis using The Cancer Genome Atlas (TCGA) data revealed a positive correlation between the NANOG signature and CD59 mRNA levels in multiple human cancer types (Supplementary Fig. S3). Taken together, we conclude that the NANOG-CD59 axis is conserved in patients with cancer and serves as a clinical determinant of disease prognosis.

### Silencing of NANOG enhances trastuzumab-induced CDC via down-regulating CD59 in NANOG^high^ tumor cells

**Figure 5:**
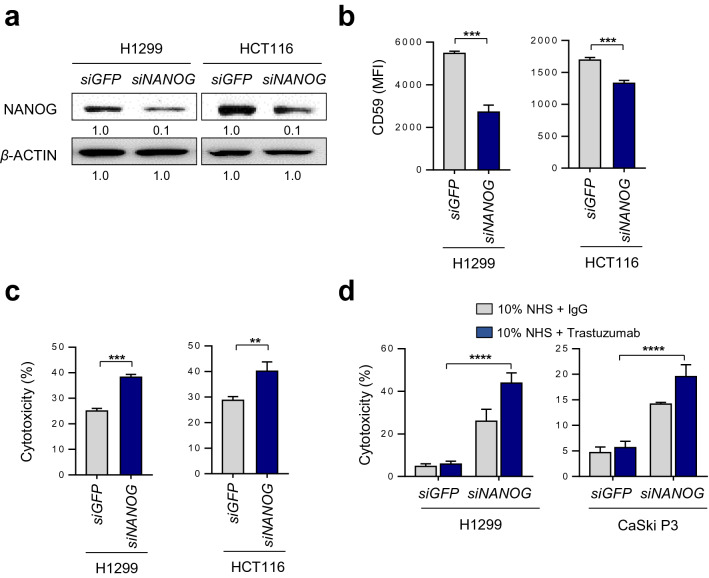
NANOG confers resistance to CDC in multiple types of human cancer cells via up-regulating CD59. (**a**–**c**) H1299 and HCT116 cells were transfected with siRNAs targeting GFP or NANOG. **(a)** The NANOG protein level was analyzed by Western blot. *β*-ACTIN was included as an internal loading control. Numbers below blot images indicate the quantitative value, as measured by fold change. Original blots were presented in Supplementary Fig. S9. **(b)** Protein levels of CD59 were determined by flow cytometry analysis. Data are presented as the mean fluorescence intensity (MFI). **(c)** CDC was measured by the LDH release assay after incubation with 20% NHS. **(d)** H1299 and CaSki P3 cells were transfected with siRNAs targeting GFP or NANOG. Trastuzumab-mediated CDC was measured by LDH assay, after incubation with 20% NHS in presence or absence of trastuzumab. All experiments were performed in triplicate, and error bars represent standard deviations from the mean. Differences in cytotoxicity or expression level were statistically tested using the Student’s t-test **(b, c)** or two-way ANOVA **(d)**: *p < 0.05; **p < 0.01, ***p < 0.001, ****p < 0.0001.

To identify the functional effects of the NANOG-CD59 axis in diverse types of human cancer cells, we further selected NANOG-up-regulated human cancer cells, such as H1299 and HCT116^13^. Knockdown of NANOG resulted in decreased expression of CD59 in the indicated cancer cells (Fig. 5a,b). Furthermore, NANOG-depleted cells were more susceptible to NHS-mediated CDC compared to cells treated with siGFP (Fig. 5c). Although recent studies have reported that complement components are activated by tumor-targeting antibodies to kill tumor cells, some therapeutic antibodies lack efficient complement-activating capacities and some tumor cells escape from CDC by high expression levels of mCRPs^20^. Given that CD59 plays roles in blocking trastuzumab-induced CDC^21,22^, we examined whether the low level of CD59 obtained by targeting NANOG can improve trastuzumab-induced CDC. To test this, we treated HER2-positive (H1299 and CaSki P3) cells^23,24^ with trastuzumab along with NHS. There was no difference in CDC of siGFP-transfected H1299 and CaSki P3 cells with or without treatment with trastuzumab, suggesting that both cells were refractory to CDC due to the presence of the NANOG-CD59 axis (Fig. 5d). Although we didn’t observe any changes in the HER2 levels upon NANOG silencing (Supplementary Fig. S4), however, NANOG depletion enhanced trastuzumab-induced CDC of H1299 and CaSki P3 cells (Fig. 5d). The result suggests that NANOG confers resistance to trastuzumab-induced CDC through the upregulation of CD59 rather than loss of HER2 antigen. Thus, our data indicate that the NANOG-CD59 axis responsible for the CDC-refractory property is conserved across multiple types of cancer cells, and that inhibition of NANOG, as part of an antibody-based therapy, represents an attractive strategy for the control of CDC-refractory cancer.

## Discussion

Cancer immunoediting drives the adaptation of tumor cells to host immune surveillance^2^. We previously found that immunoediting driven by CTLs eventually leads to the enrichment of tumor cells refractory to multiple clinical interventions, including immunotherapy^4^. Here, we showed that CTL-mediated immune pressure triggers cross-resistance of tumor cells to the complement system, a part of the innate immune system. In doing so, we discovered that the TF NANOG plays a crucial role in cross-resistance to CDC during CTL-mediated immunoediting of tumor cells.

As the complement pathway participates in all facets of immune surveillance by collaborating with both the innate and adaptive immune systems, cancer cells actively escape complement and immune surveillance by upregulating mCRPs^25^. Although the upregulation of mCRPs has been reported in multiple human cancers^26^, its regulatory mechanism, particularly in the course of cancer immunoediting, has not yet been extensively studied. In this respect, we noted that immune pressure imposed by antigen (Ag)-specific CTLs drives the acquisition of NANOG, a master TF that mediates the emergence of a stem-like cancer cell state and immune evasion^16^. Notably, we showed that the CDC-refractory phenotypes over the course of CTL-mediated immune selection are closely linked to an expression state of NANOG and mCRPs, especially CD59. In this study, we demonstrated that CD59 is a novel NANOG transcriptional target, suggesting that NANOG facilitates the escape of complement attack by directly upregulating CD59 expression.

Studies have reported that mCRPs, including CD59, were associated with disease progression, chemoresistance, and metastatic potential in multiple human cancers^27,28^. Notably, the level of CD59 within the tumor was strongly correlated with disease progression and survival in cervical cancer patients. Furthermore, the NANOG-CD59 axis was widely conserved in various TCGA cohorts and in tumor tissue from patients with cervical cancer. These data suggest that the expression status of CD59 (either alone or in conjunction with NANOG) within the tumor tissue may serve as a potential prognostic marker. The crucial role of NANOG in multiple aggressive phenotypes, including multi-modal resistance and stem-like property of cancer cells^14^, raises an obvious question whether CD59 can contribute to NANOG-mediated aggressive phenotypes of tumor cells. Interestingly, although inhibition of CD59 leads to a significant increase in the susceptibility of CaSki-NANOG cells to NHS-mediated CDC, CD59 did not influence the hyperactivation of AKT signaling, which is essential for immune resistance to CTL killing as well as stem-like property of NANOG^+^ tumor cells^13^ and the cancer stem cell (CSC)-like property (Supplementary Fig. S5). In light of this, our data propose that increase in CD59 might result from immune selection of NANOG^+^ CTL-refractory tumor cells, but it may not be its cause, at least during CTL-mediated immune selection.

The complement is an important player in Ab-induced tumor cell death, and therefore, it has a major impact on the efficacy of therapeutic IgG1 monoclonal Abs, such as trastuzumab^29^. However, highly expressed mCRPs may block the complement pathway and thus protect tumor cells from mAb-induced CDC^30^. Consistent with the results, we showed that a NANOG^high^ tumor with CTL-refractory property exhibited resistance to trastuzumab-mediated CDC, whereas silencing of NANOG led to increase in susceptibility of trastuzumab-mediated CDC. Furthermore, recent preclinical and clinical data indicate that avelumab, a fully human IgG1 anti-PD-L1 monoclonal antibody (mAb) with potential Ab-mediated CDC, can be safely administered to cancer patients with a toxicity profile comparable to other mAbs and without lysis of PD-L1-positive activated immune cells. This antibody yielded durable responses in a phase II trial in advanced Merkel cell carcinoma patients^31^. On the basis of our findings, tumors with a high level of NANOG could be resistance to avelumab-mediated CDC. Therefore, our results propose a rationale whereby strategies blockading NANOG-CD59 axis in immune-edited tumors could enhance the efficacy of therapeutic mAbs against tumors by promoting Ab-mediated CDC.

Taken together, our present study revealed a possible mechanism through which selection imposed by T-cell based immunotherapy triggered complement-resistant phenotypes in the TME. Critically, NANOG^+^ tumor cells enriched through selective pressure imposed by CTLs preferentially expressed CD59 via transcriptional regulation, thereby promoting the CDC-refractory phenotype. We believe these results hold important implications for the clinical application of a CDC-mediated therapeutic antibody.

## Materials and methods

### Cells

CaSki, H1299, HCT116, and HEK293 cells were purchased from American Type Culture Collection (ATCC). All cell lines were obtained between 2010 and 2014, and tested for mycoplasma using Mycoplasma Detection Kit (Thermo Fisher Scientific, San Jose, CA, USA). The identities of cell lines were confirmed by short tandem repeat (STR) profiling by IDEXX Laboratories Inc. and used within 6 months for testing. Human immune-resistant CaSki P3 cells were previously established^17^. CaSki-no insert and CaSki-NANOG stable cell lines were generated by retroviral transduction with the pMSCV-no insert and pMSCV-NANOG^13^. H1299, HCT116, and HEK293 cells were cultured in Dulbecco’s Modified Eagle Medium (DMEM) (Welgene #LM001-05) containing 100 units/ml of penicillin–streptomycin and 10% fetal bovine serum (FBS). CaSki cells were cultured in RPMI1640 containing 100 units/ml of penicillin–streptomycin and 10% FBS. All cells were grown at 37 °C in a 5% CO_2_ incubator/humidified chamber.

### DNA constructs

Plasmid DNA constructs of FLAG-tagged NANOG WT and NANOG MUT were previously generated^13^. The promoter region of the CD59 gene was isolated by polymerase chain reaction (PCR) from genomic DNA extracted from CaSki cells using the primer set, 5’-CGGGTACCAGGAGACATGCTTTAAATATC-3’ (forward) and 5’-AACTCGAGCCGCTTCTGCGCTCAG-3’ (reverse). The PCR products were digested with *Kpn*I and *Xho*I and subcloned into the *Kpn*I/*Xho*I restriction sites of the pGL3-Basic vector (Promega).

### siRNA constructs

Synthetic small interfering RNAs (siRNAs) specific for green fluorescent protein (GFP), NANOG, CD46, CD55, and CD59 were purchased from Bioneer (Korea); Non-specific GFP, 5’-GCAUCAAGGUGAACUUCAA-3’ (sense), 5’-UUGAAGUUCACCUUGAUGC-3’ (antisense); NANOG^15^, 5’-CUAAACUACUCCAUGAACA-3’ (sense), 5’-UGUUCAUGGAGUAGUUUAG-3’ (antisense); CD46, 5’-CACCUUUAGUGAAGUAGAA-3’ (sense), 5’-UUCUACUUCACUAAAGGUG-3’ (antisense); CD55, 5’-GUCUCACCAACUUCUCAGA-3’ (sense), 5’-UCUGAGAAGUUGGUGAGAC-3’ (antisense); CD59, 5’-CUCCAAUGACCACCUACUA-3’ (sense), 5’-UAGUAGGUGGUCAUUGGAG-3’ (antisense).

### Real-time quantitative RT-PCR

The RNA purification and quantitative reverse transcriptase polymerase chain reaction (qRT-PCR) were performed, as described previously^4^. Real-time quantitative PCR was performed using iQ SYBR Green super mix (Bio-Rad) with the following specific primers: CD46, 5’-ATACCTCCTCTTGCCACCCATAC-3’ (forward) and 5’-GTCACCACAATAAATCGTGCTCT-3’ (reverse); CD55, 5’-ATCCCTCAAACAGCCTTAT-3’ (forward) and 5’-CCATTTCGTATTTCTCCC-3’ (reverse); CD59, 5’-CTAACCCAACTGCTGACTG-3’ (forward) and 5’-CTGATAAGGATGTCCCACC-3’ (reverse); β-ACTIN, 5’-CGACAGGATGCAGAAGGAGA-3’ (forward) and 5’-TAGAAGCATTTGCGGTGGAC-3’ (reverse) on a CFX96 real-time PCR detection system. The specificity of each primer set was determined by the melting curve analysis. All real-time quantitative PCR experiments were performed in triplicate, and quantification cycle (Cq) values were determined using Bio-Rad CFX96 Manager 3.0 software. Relative quantification of the mRNA levels was performed using the comparative Ct method with β-ACTIN as the reference gene.

### Flow cytometry analysis

To detect the expression of CD46, CD55, or CD59, flow cytometry analysis was performed, as described previously^15^. The collected cells were reacted with FITC-conjugated anti-CD46, anti-CD55 or anti-CD59 Abs (Medical & Biological Laboratories) for 1 h at 4 °C. Stained cells were washed twice and analyzed using a FACSVerse flow cytometer (BD Biosciences). Data acquisition was performed on a FACSVerse flow cytometer (BD Biosciences) with BD FACSuite software.

### Western blot analysis

Western blotting was performed, as described previously^15^. Primary antibodies against NANOG (A300-397A, Bethyl Laboratories, Montgomery, TX)^4^ and β-ACTIN (M177-3, Medical and Biological Laboratories)^4^ were used for Western blotting, followed by the appropriate secondary antibodies conjugated with horseradish peroxidase. Some membranes were stripped by treatment with WB stripping Solution (Thermo Fisher Scientific, USA). Immunoreactive bands were developed with the chemiluminescence ECL detection system (Elpis Biotech, Daejeon, Korea), and signals were detected using a luminescent image analyzer (LAS-4000 Mini, Fujifilm, Tokyo). The intensity of the western blot signals was quantified using Multi-gauge software (Fujifilm).

### Lactate dehydrogenase (LDH) release assay

For estimating CDC, LDH release assay was performed using a CytoTox 96®Non-Radioactive Cytotoxicity Assay Kit (Promega), as described previously^32^. The normal human serum (NHS, Sigma Aldrich, St. Louis, MO, USA) was used as source of complement and natural antibodies. Briefly, the cells were incubated with NHS that was diluted with opti-MEM in the presence or the absence of trastuzumab, an anti-HER2 antibody. The plate was incubated for 4 h at 37 °C, and then the released LDH was measured.

### Luciferase assay

To determine the CD59 promoter activity, luciferase assay was performed, as described previously^4^. Cells were transfected with 100 ng of pGL3-CD59 reporter, and 100 ng of empty vector, NANOG WT or NANOG MUT, together with 20 ng of CMV/β-galactosidase plasmid to normalize the transfection efficiency.

### Chromatin immunoprecipitation (ChIP) and quantitative ChIP (qChIP) assays

The ChIP kit (Millipore) was employed according to the manufacturer’s instructions, as described previously^4^. For qChIP assay, immunoprecipitated DNA was quantified by real-time qPCR using the following primer sets: N1, 5’-ATACCAGGATTTGAGCACCACC-3’ (forward) and 5’-AACTGTCTTGACGCTTCTACTG-3’ (reverse); N2, 5’-AACAGTAGAAGCGTCAAGACA-3’ (forward) and 5’-TTACATCTCAGGGCTGCTTGT-3’ (reverse).

### Tissue samples and immunohistochemistry

Tissue microarrays (TMAs) constructed from a cohort of 386 formalin-fixed, paraffin-embedded tumor specimens and matched nonadjacent normal specimens, have been described previously. The study samples from 178 cervical cancer patients and 126 cervical intraepithelial neoplasia (CIN) patients obtained by surgical resection in Gangnam Severance Hospital between 1996 and 2010 were histologically confirmed by a pathologist. Some of the paraffin blocks were provided by the Korea Gynecologic Cancer Bank through the Bio & Medical Technology Development Program of the Ministry of Education, Science and Technology, Korea (NRF-2017M3A9B8069610). Tissue samples were collected from patients who had signed informed consent form. This study was approved by the Institutional Review Board of Gangnam Severance Hospital (IRB# 3-2014-0184; Seoul, South Korea) and was additionally approved by the Office of Human Subjects Research at the National Institutes of Health. All procedures were conducted in accordance with the guidelines of the Declaration of Helsinki.

Immunohistochemistry was performed on 5-μm sections of the TMA using a detailed procedure described previously^19^. After deparaffinization and rehydration, heat-induced antigen retrieval was performed for 20 min using antigen retrieval pH 6.0 (Dako, Carpinteria, CA, USA). Endogenous peroxidase activity was quenched with 3% H2O2 for 10 min. The sections were incubated at room temperature with mouse polyclonal anti-CD59 antibodies (Santa Cruz Biotechnology, clone H-85; Dallas, TX, USA) at 1:500 dilution for 60 min. The antigen–antibody reaction was detected with EnVision + Rabbit-HRP (Dako) and visualized with DAB (3,3-diaminobenzadine; Dako). Negative controls including immunoglobulin G (IgG) and omission of the primary antibody were concurrently performed, and the TMA included appropriate positive control tissues. Finally, tissue sections were lightly counterstained with hematoxylin and then examined by light microscopy. NANOG protein expression was previously evaluated in the same cohort^19^. Immunohistochemistry scoring (histoscore) was performed using Visiopharm Digital Image Analysis (DIA) software v2020_01_14 (Visiopharm, Hørsholm, Hørsholm, Denmark). In brief, bule-colored tumor nuclei were initially defined, and then brown-colored (DAB) nuclei and cytoplasm separated spectrally (Supplementary Fig. S6). The DAB intensity was categorized as 0 (negative), 1 + (weak), 2 + (moderate), and 3 + (strong) according to the distribution pattern across cores. The final score was calculated by multiplying the DAB staining intensity and the percentage of positive cells as described previously^19^.

### Statistical analysis

All data are representative of at least three separate experiments. Statistical differences were calculated by either the student’s t-test (two-tailed, unpaired), one-way ANOVA, or two-way ANOVA using GraphPad Prism software. For tissue samples and immunohistochemistry, statistical analyses were performed using IBM SPSS statistics version 21 (IBM Corporation, Armonk, NY, USA). Statistical comparisons of the differences in protein expressions in different groups were performed using non-parametric statistics (Kruskal–Wallis and Mann–Whitney U), where appropriate. The Chi-square test was used to perform statistical comparisons of categorical variables. Correlation between protein expressions was determined by Spearman's rank correlation. The Kaplan–Meier method with the log-rank test was used for the estimation of survival distributions. Results with two-tailed p values less than 0.05 were considered statistically significant.

## Supplementary Information

Supplementary Information.

## Data Availability

Transcriptome data from TCGA were deposited into GEPIA2 data repository portal (http://gepia2.cancer-pku.cn/).

## References

[1] Gajewski TF, Schreiber H, Fu YX (2013). Innate and adaptive immune cells in the tumor microenvironment. Nat. Immunol..

[2] Galon J, Bruni D (2020). Tumor immunology and tumor evolution: intertwined histories. Immunity.

[3] Song KH (2018). Mitochondrial reprogramming via ATP5H loss promotes multimodal cancer therapy resistance. J. Clin. Invest..

[4] Song KH (2020). HSP90A inhibition promotes anti-tumor immunity by reversing multi-modal resistance and stem-like property of immune-refractory tumors. Nat. Commun..

[5] Schreiber RD, Old LJ, Smyth MJ (2011). Cancer immunoediting: integrating immunity's roles in cancer suppression and promotion. Science.

[6] Merle NS, Noe R, Halbwachs-Mecarelli L, Fremeaux-Bacchi V, Roumenina LT (2015). Complement system part II: role in immunity. Front. Immunol..

[7] Geller A, Yan J (2019). The role of membrane bound complement regulatory proteins in tumor development and cancer immunotherapy. Front. Immunol..

[8] Reis ES, Mastellos DC, Ricklin D, Mantovani A, Lambris JD (2018). Complement in cancer: untangling an intricate relationship. Nat. Rev. Immunol..

[9] Sorman A, Zhang L, Ding Z, Heyman B (2014). How antibodies use complement to regulate antibody responses. Mol. Immunol..

[10] Macor P (2007). In vivo targeting of human neutralizing antibodies against CD55 and CD59 to lymphoma cells increases the antitumor activity of rituximab. Cancer Res..

[11] Rosner T, Lohse S, Peipp M, Valerius T, Derer S (2014). Epidermal growth factor receptor targeting IgG3 triggers complement-mediated lysis of decay-accelerating factor expressing tumor cells through the alternative pathway amplification loop. J. Immunol..

[12] Mao CP, Wu T, Song KH, Kim TW (2014). Immune-mediated tumor evolution: nanog links the emergence of a stem like cancer cell state and immune evasion. Oncoimmunology.

[13] Noh KH (2012). Nanog signaling in cancer promotes stem-like phenotype and immune evasion. J. Clin. Invest..

[14] Song KH (2017). HDAC1 upregulation by NANOG promotes multidrug resistance and a stem-like phenotype in immune edited tumor cells. Cancer Res..

[15] Kim S (2021). LC3B upregulation by NANOG promotes immune resistance and stem-like property through hyperactivation of EGFR signaling in immune-refractory tumor cells. Autophagy.

[16] Oh SJ (2020). Far beyond cancer immunotherapy: reversion of multi-malignant phenotypes of immunotherapeutic-resistant cancer by targeting the NANOG signaling axis. Immune Netw..

[17] Lee YH (2015). Gain of HIF-1alpha under normoxia in cancer mediates immune adaptation through the AKT/ERK and VEGFA axes. Clin. Cancer Res. Off. J. Am. Assoc. Cancer Res..

[18] Mamidi S (2015). Neutralization of membrane complement regulators improves complement-dependent effector functions of therapeutic anticancer antibodies targeting leukemic cells. Oncoimmunology.

[19] Oh SJ (2018). Targeting cyclin D-CDK4/6 sensitizes immune-refractory cancer by blocking the SCP3-NANOG axis. Cancer Res..

[20] Gorter A (1996). Expression of CD46, CD55, and CD59 on renal tumor cell lines and their role in preventing complement-mediated tumor cell lysis. Lab. Invest. J. Tech. Methods Pathol..

[21] Wang Y (2017). CD55 and CD59 expression protects HER2-overexpressing breast cancer cells from trastuzumab-induced complement-dependent cytotoxicity. Oncol. Lett..

[22] Bellone S (2012). Downregulation of membrane complement inhibitors CD55 and CD59 by siRNA sensitises uterine serous carcinoma overexpressing Her2/neu to complement and antibody-dependent cell cytotoxicity in vitro: implications for trastuzumab-based immunotherapy. Br. J. Cancer.

[23] Ahmed N (2015). Human epidermal growth factor receptor 2 (HER2)—specific chimeric antigen receptor-modified T cells for the immunotherapy of HER2-positive sarcoma. J. Clin. Oncol. Off. J. Am. Soc. Clin. Oncol..

[24] Meira DD (2009). Combination of cetuximab with chemoradiation, trastuzumab or MAPK inhibitors: mechanisms of sensitisation of cervical cancer cells. Br. J. Cancer.

[25] Kleczko EK, Kwak JW, Schenk EL, Nemenoff RA (2019). Targeting the complement pathway as a therapeutic strategy in lung cancer. Front. Immunol..

[26] Fishelson Z, Donin N, Zell S, Schultz S, Kirschfink M (2003). Obstacles to cancer immunotherapy: expression of membrane complement regulatory proteins (mCRPs) in tumors. Mol. Immunol..

[27] Hsu YF (2010). Complement activation mediates cetuximab inhibition of non-small cell lung cancer tumor growth in vivo. Mol. Cancer.

[28] Hu W (2011). Human CD59 inhibitor sensitizes rituximab-resistant lymphoma cells to complement-mediated cytolysis. Cancer Res..

[29] Spiridon CI (2002). Targeting multiple Her-2 epitopes with monoclonal antibodies results in improved antigrowth activity of a human breast cancer cell line in vitro and in vivo. Clin. Cancer Res. Off. J. Am. Assoc. Cancer Res..

[30] Coral S (2000). Overexpression of protectin (CD59) down-modulates the susceptibility of human melanoma cells to homologous complement. J. Cell. Physiol..

[31] Hamilton G, Rath B (2017). Avelumab: combining immune checkpoint inhibition and antibody-dependent cytotoxicity. Exp. Opin. Biol. Therapy.

[32] Song KH (2010). Cloning and functional characterization of pig CMP-N-acetylneuraminic acid hydroxylase for the synthesis of N-glycolylneuraminic acid as the xenoantigenic determinant in pig-human xenotransplantation. Biochem. J..

