# Supplementary material for: NANOG confers resistance to complement-dependent cytotoxicity in immune-edited tumor cells through up-regulating CD59
Source: Sci Rep. 2022 May 23;12:8652. doi: 10.1038/s41598-022-12692-6 (PMC9126891; doi:10.1038/s41598-022-12692-6)
Supplement: Supplementary file 1 — Supplementary Information. [file 41598_2022_12692_MOESM1_ESM.pdf]

Supplementary Information

NANOG confers resistance to complement-dependent cytotoxicity in immune-edited tumor cells through up-regulating CD59

Supplementary Table S1. Clinicopathologic significance of CD59 protein expression in human cervical cancers

Supplementary Figure S1. Knockdown efficiencies of target proteins by designed siRNAs.

Supplementary Figure S2. Kaplan-Meier plots of disease-free survival for cervical cancer patients according to CD59 level.

Supplementary Figure S3. Correlation plot of CD59 and NANOG signature in pan-tumor types.

Supplementary Figure S4. Silencing of NANOG does not affect HER2 level in H1299 and CaSki P3 cells.

Supplementary Figure S5. Silencing of CD59 does not affect the CSC-like property and AKT signaling of CaSki-NANOG cells.

Supplementary Figure S6. Digital image analysis of immunohistocal staining.

Supplementary Figure S7. Whole Western blot from Figure 2a.

Supplementary Figure S8. Whole Western blot from Figure 2c.

Supplementary Figure S9. Whole Western blot from Figure 5a.

Supplementary Figure S10. Whole Western blot from Supplementary Figure S5b.

Supplementary Table S1. Clinicopathologic significance of CD59 protein expression in human cervical cancers

	CD59		
	No.	Mean IHC score (95% CI)	*p value*
**All study subjects**	386	124 (116-132)	
**Diagnostic category**			*<0.001*
Normal	82	39 (32-46)	
Low grade CIN	43	53 (36-70)	
High grade CIN	83	120 (107-134)	
Cancer	178	182 (174-190)	
**Age**			*0.008*
≤ 50	107	174 (163-184)	
> 50	71	194 (184-205)	
**FIGO stage**			*0.047*
I - IIA	136	178 (170-185)	
IIB - IV	42	196 (174-217)	
**Cell type**			*0.985*
SCC	148	182 (173-191)	
Others	30	182 (165-198)	
**Tumor grade**			*0.387*
Well + Moderate	107	182 (172-192)	
Poor	57	189 (176-202)	
**Tumor size**			*0.053*
< 4 cm	125	177 (169-185)	
≥ 4 cm	53	193 (177-210)	
**LN metastasis**			*0.844*
Negative	115	173 (164-182)	
Positive	31	175 (153-197)	
**Chemoradiation response**			*0.320*
Good	48	182 (167-197)	
Bad	21	196 (168-224)	

SCC, squamous cell carcinoma; FIGO, International Federation of Gynecology and Obstetrics; LN metastasis, Lymph node metastasis. Protein expression was determined through analysis of an immunohistochemically stained tissue array, as described in the materials and methods section. The Mann-Whitney U test was used to characterize relationship between categorical variables.

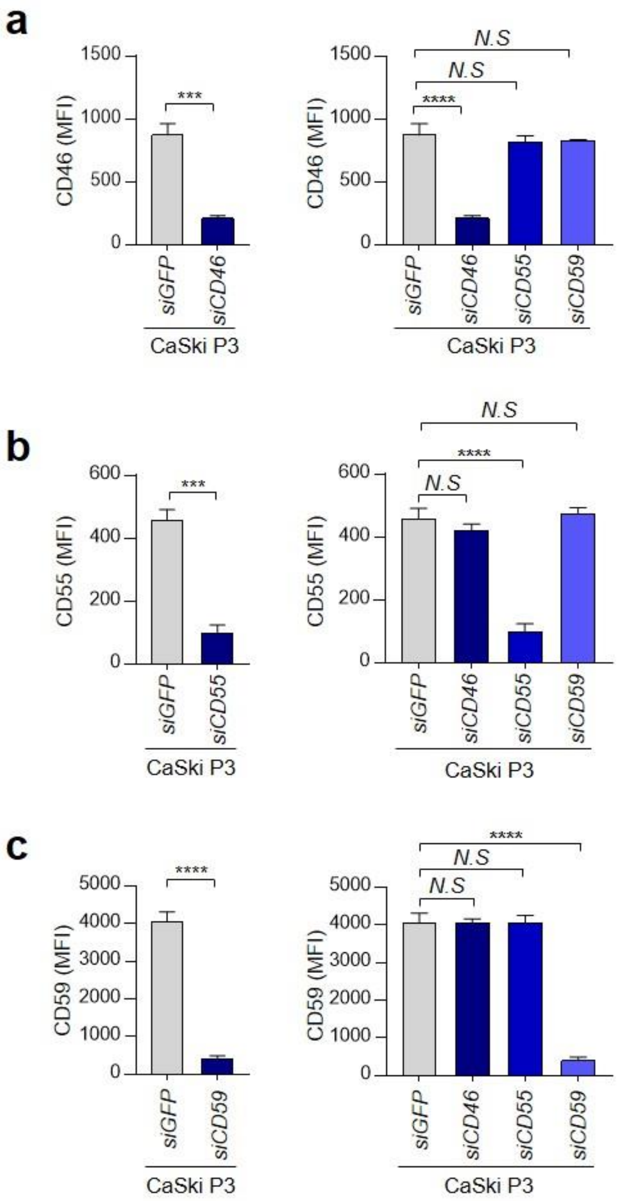

Supplementary Figure S1. Knockdown efficiencies of target proteins by designed siRNAs. CaSki P3 cells were transfected with siRNAs targeting GFP, CD46, CD55, or CD59. **(a-c)** Protein levels of mCRPs were determined by staining with CD46, CD55, or CD59, followed by flow cytometry analysis. Data are presented as the mean fluorescence intensity (MFI). All experiments were performed in triplicate, and error bars represent standard deviations from the mean. **(a-c)** Differences in expression levels were statistically tested using the Student's t-test or one-way ANOVA: **, $p < 0.01$, ***, $p < 0.001$, ****, $p < 0.0001$, *NS*, not significant.

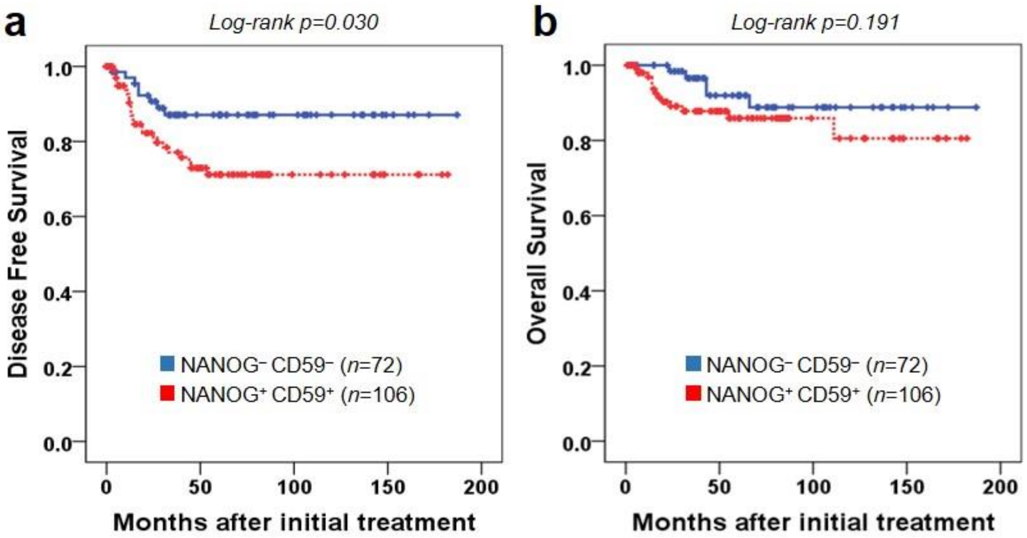

Supplementary Figure S2. Kaplan-Meier plots of disease-free survival for cervical cancer patients according to CD59 level. (a) High level of CD59 was associated with poor disease-free survival (p < 0.030). **(b)** High level of CD59 exhibited tendency of short overall survival. p-values by Log-rank (Mantel-Cox) test are indicated **(a and b)**.

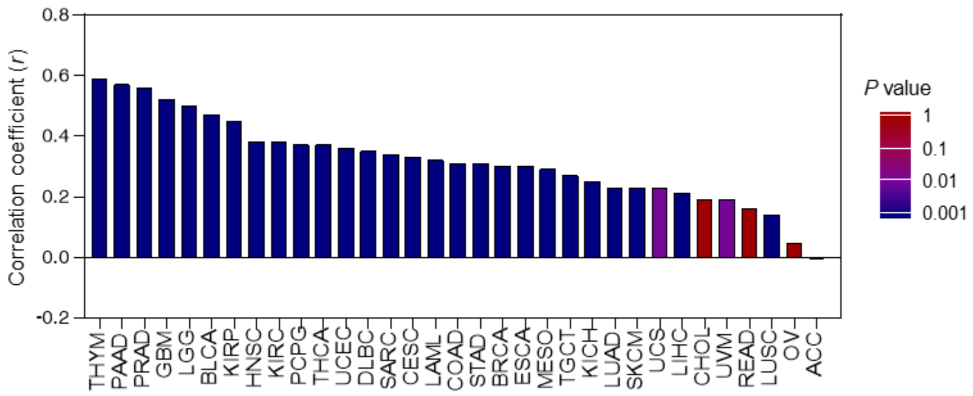

Supplementary Figure S3. Correlation plot of CD59 and NANOG signature in pan-tumor types. Correlation between NANOG signature gene expression and CD59 expression in indicated various type of cancer patients from The Cancer Genome Atlas (TCGA) data organized in the Gene Expression Profiling Interactive Analysis (GEPIA2) website.

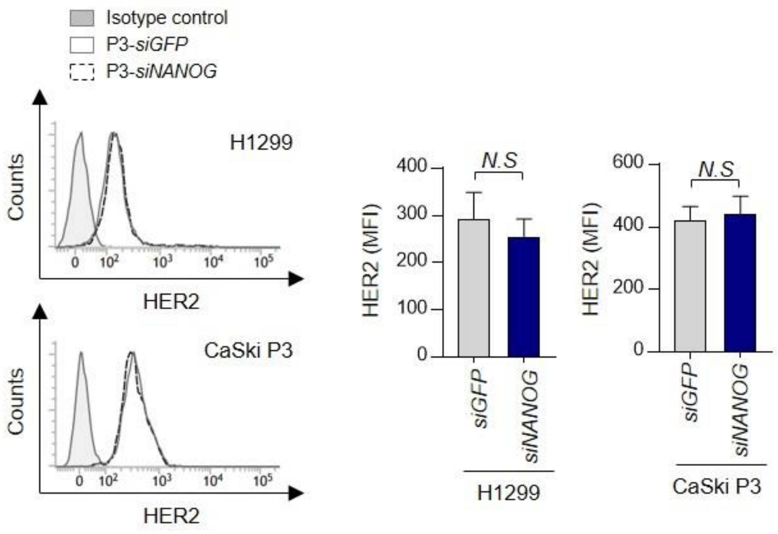

Supplementary Figure S4. Silencing of NANOG does not affect HER2 level in H1299 and CaSki P3 cells. H1299 and CaSki P3 cells were transfected with siRNAs targeting GFP or NANOG. Protein levels of HER2 were determined by flow cytometry analysis. Data are presented as the mean fluorescence intensity (MFI). All experiments were performed in triplicate, and error bars represent standard deviations from the mean. Differences in expression level were statistically tested using the Student's t-test: ***, $p < 0.001$, *NS*, not significant.

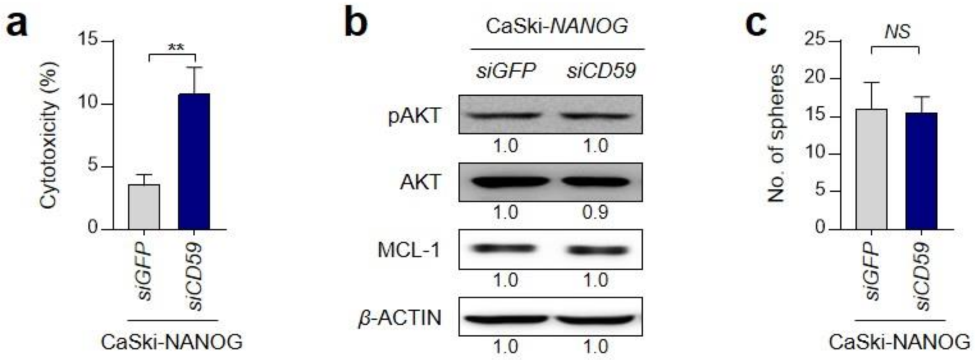

Supplementary Figure S5. Silencing of CD59 does not affect the CSC-like property and AKT signaling of CaSki-NANOG cells. CaSki-NANOG cells were transfected with siRNAs targeting GFP or CD59. **(a)** CDC was measured by the LDH release assay after incubation with 20% NHS. **(b)** Phospho-AKT, AKT, and MCL-1 expressions were analyzed by immunoblotting. β-ACTIN was included as an internal loading control. Numbers below blots indicate the expression, as measured by fold change. Original blots were presented in Supplementary Fig. S7 **(c)** The degree of CSC-like phenotypes was assessed by the sphere-forming capacity in low-density suspension culture. All experiments were performed in triplicate, and error bars represent standard deviations from the mean. Differences in cytotoxicity or expression level were statistically tested using the Student's t-test: **, $p < 0.01$, *NS*, not significant.

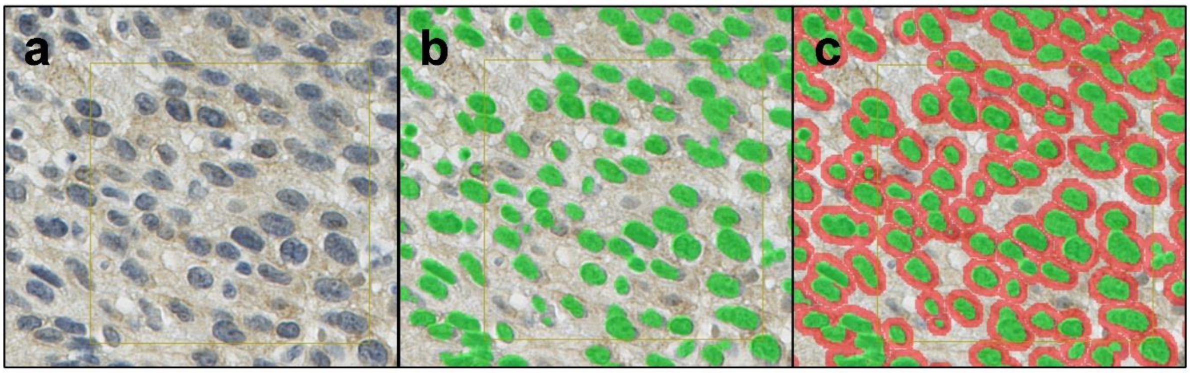

Supplementary Figure S6. Digital image analysis of immunohistocal staining. With the original image **(a)**, nucleus (green) **(b)** and cytoplasm (orange) **(c)** are classified, and the mean intensity for each fields are measured. The final histoscore was calculated by multiplying the intensity and percentage of staining resulting in a range of 0 to 300.

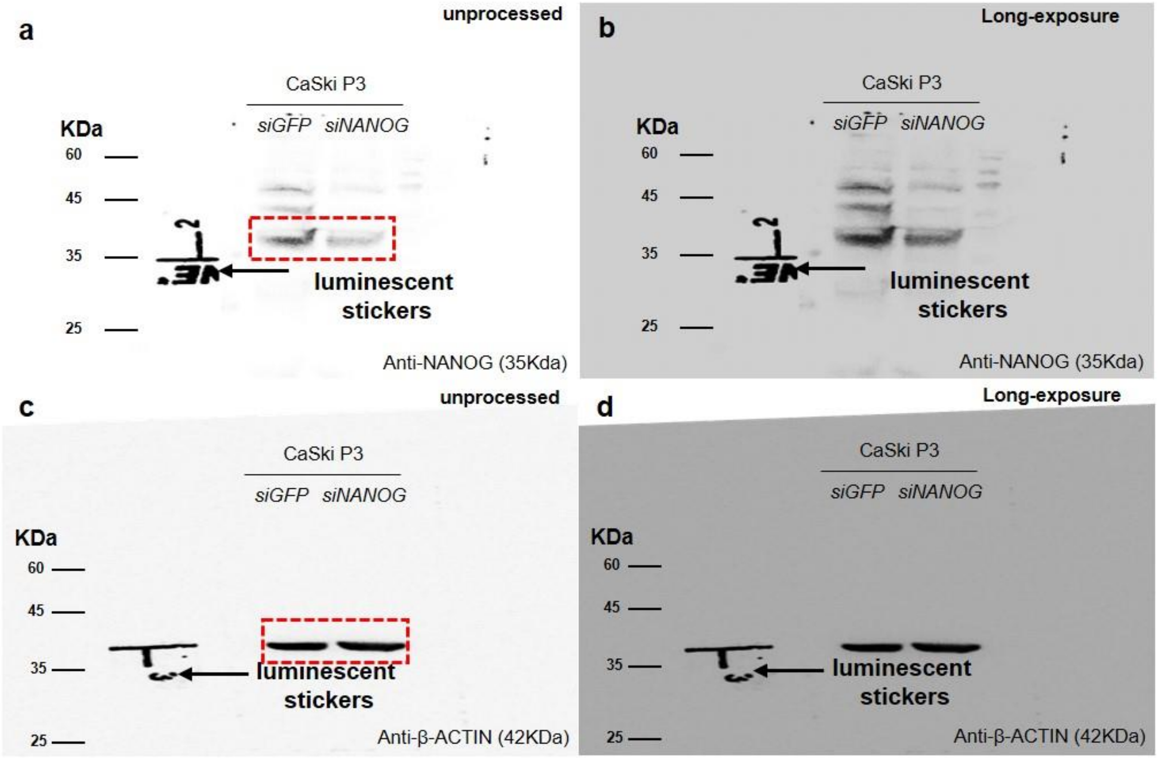

Supplementary Figure S7. Whole Western blot from Figure 2a.

Original Western blots of NANOG **(a, b)** and β-ACTIN **(c, d)** at exposure times that gave non-saturated signals used for quantification. The membrane **(a, c)** represented unprocessed original image data, and membrane **(b, d)** was long-exposure of original membrane **(a, c)**. The red dotted boxes indicate the cropped area shown in Fig. 2a. The presented original western images obtained from blotting of cutted membrane with only the necessary lanes but not of the full membrane. The glowscale autoradiograph marker (luminescent stickers) was used to indicate the size corresponding the protein. NANOG bands of membrane (a) was stripped by treatment with WB stripping Solution (Thermo scientific, USA) and reproved with anti-β-actin antibody for confirmation of equivalence of the loading protein in each lane.

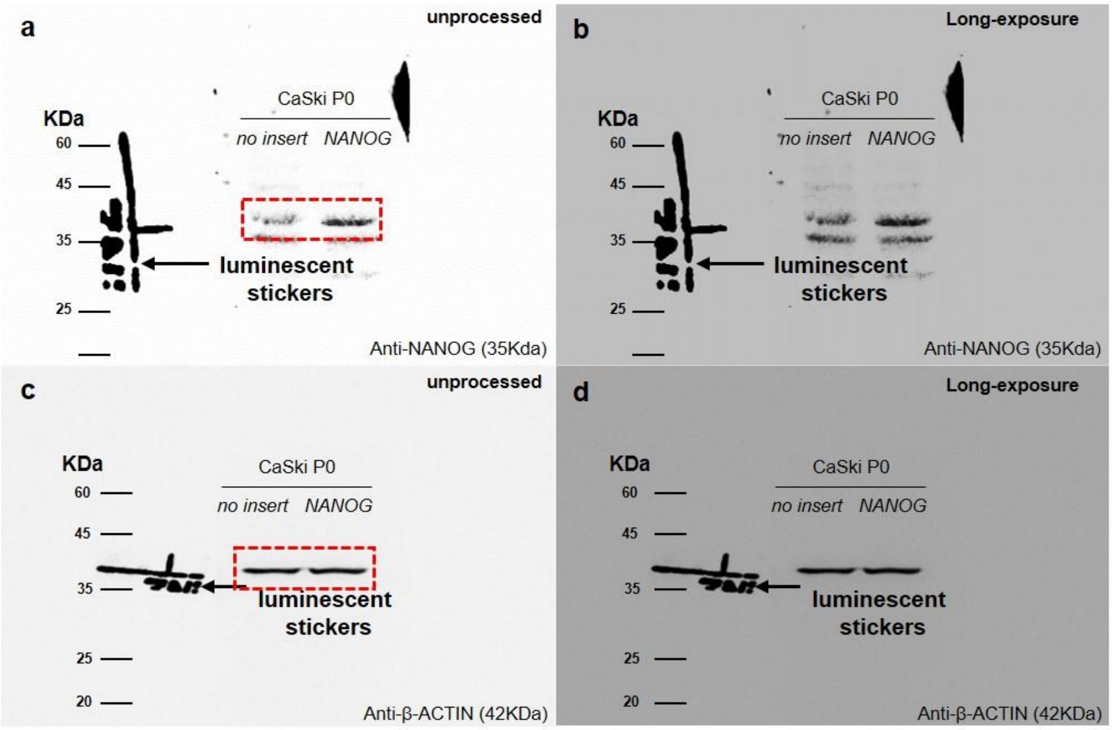

Supplementary Figure S8. Whole Western blot from Figure 2c.

Original Western blots of NANOG **(a, b)** and β-ACTIN **(c, d)** at exposure times that gave non-saturated signals used for quantification. The membrane **(a, c)** represented unprocessed original image data, and membrane **(b, d)** was long-exposure of original membrane **(a, c)**. The red dotted boxes indicate the cropped area shown in Fig. 2c. The presented original western images obtained from blotting of cutted membrane with only the necessary lanes but not of the full membrane. The glowscale autoradiograph marker (luminescent stickers) was used to indicate the size corresponding the protein. NANOG bands of membrane **(a)** was stripped by treatment with WB stripping Solution (Thermo scientic, USA) and reproved with anti-β-actin antibody for confirmation of equivalence of the loading protein in each lane.

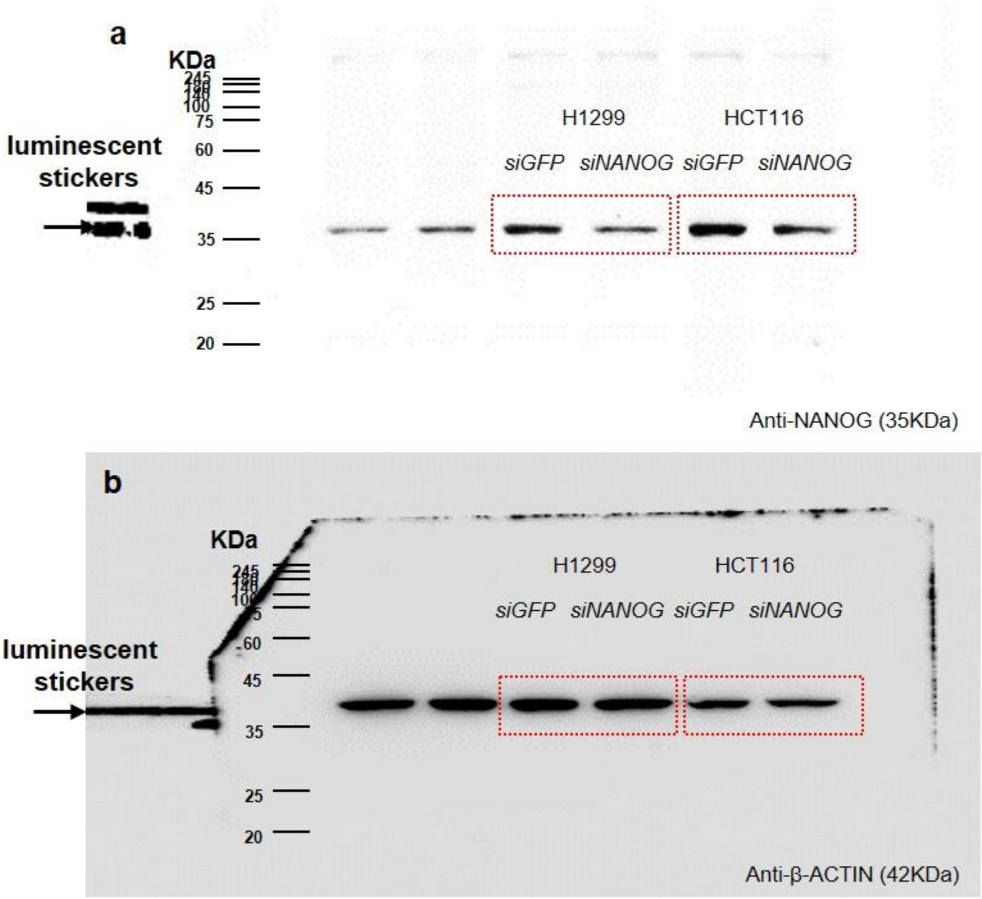

Supplementary Figure S9. Whole Western blot from Figure 5a.

Full-length Western blots of NANOG **(a)** and β-ACTIN **(b)** at exposure times that gave non-saturated signals used for quantification. The red dotted boxes indicate the cropped area shown in Fig. 5a. The glowscale autoradiograph marker (luminescent stickers) was used to indicate the size corresponding the protein. NANOG bands of membrane **(a)** was stripped by treatment with WB stripping Solution (Thermo scientic, USA) and reproved with anti-β-actin antibody for confirmation of equivalence of the loading protein in each lane.

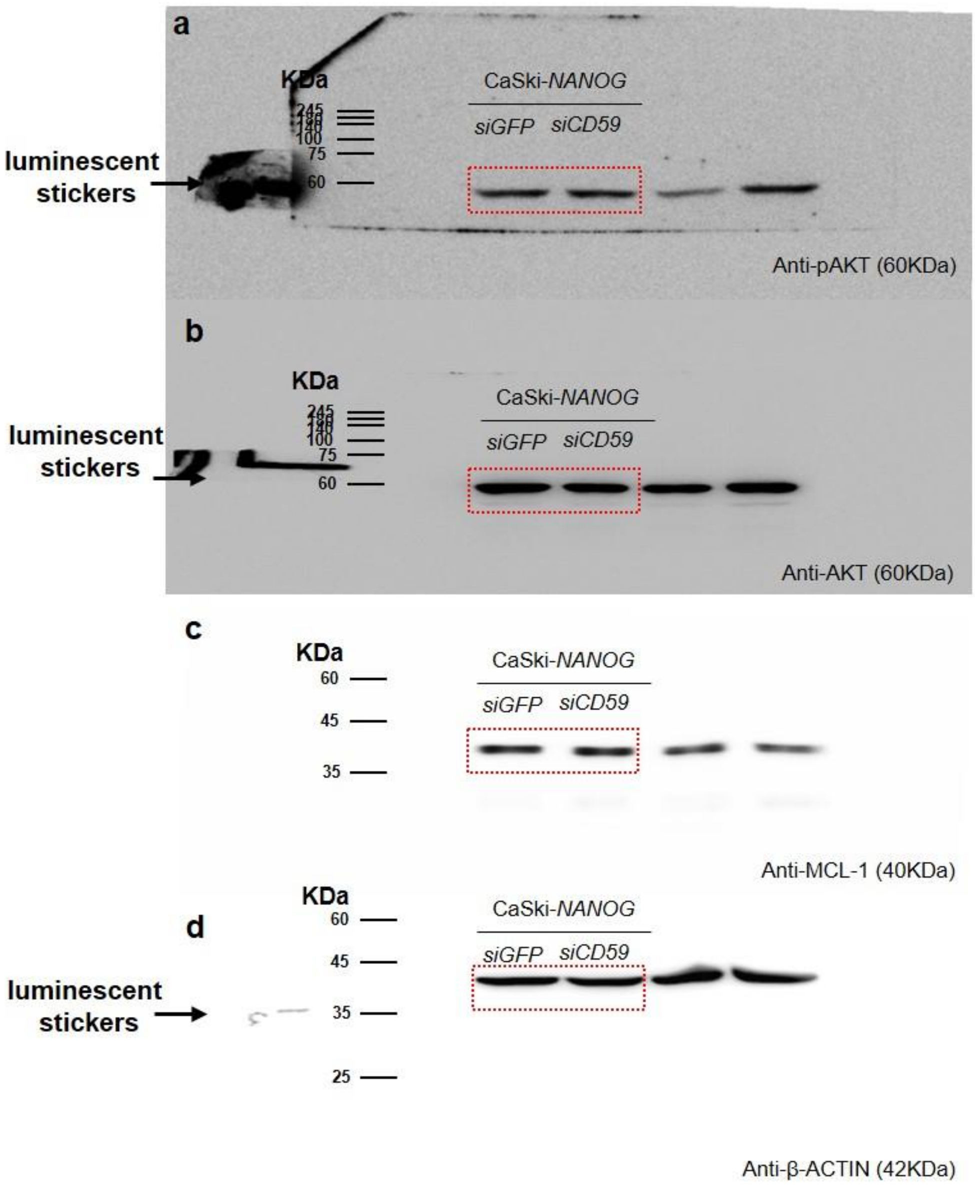

Supplementary Figure S10. Whole Western blot from Supplementary Figure S5b.

Original Western blots of pAKT **(a)**, AKT **(b)**, MCL-1 **(c)** and β-ACTIN **(d)** at exposure times that gave non-saturated signals used for quantification. The presented original western images obtained from blotting of cutted membrane with only the necessary lanes but not of the full membrane. The red dotted boxes indicate the cropped area shown in Supplementary Fig. S5b. The glowscale autoradiograph marker (luminescent stickers) was used to indicate the size corresponding the protein. The blot for anti-pAKT antibody **(a)** or anti-MCL-1 antibody **(c)** was stripped and reproved with anti-AKT antibody **(b)** or anti-β-actin antibody **(d)** as a loading control, respectively.
